# Supplementary material for: Effectiveness and Safety of Ayurvedic Medicines in Type 2 Diabetes Mellitus Management: A Systematic Review and Meta-Analysis
Source: Front Pharmacol. 2022 Jun 8;13:821810. doi: 10.3389/fphar.2022.821810 (PMC9213670; doi:10.3389/fphar.2022.821810)
Supplement: Supplementary file 1 [file DataSheet1.zip › Supplementary Material/Forest and Funnel Plots/Tinospora cordifolia/TC.pdf]

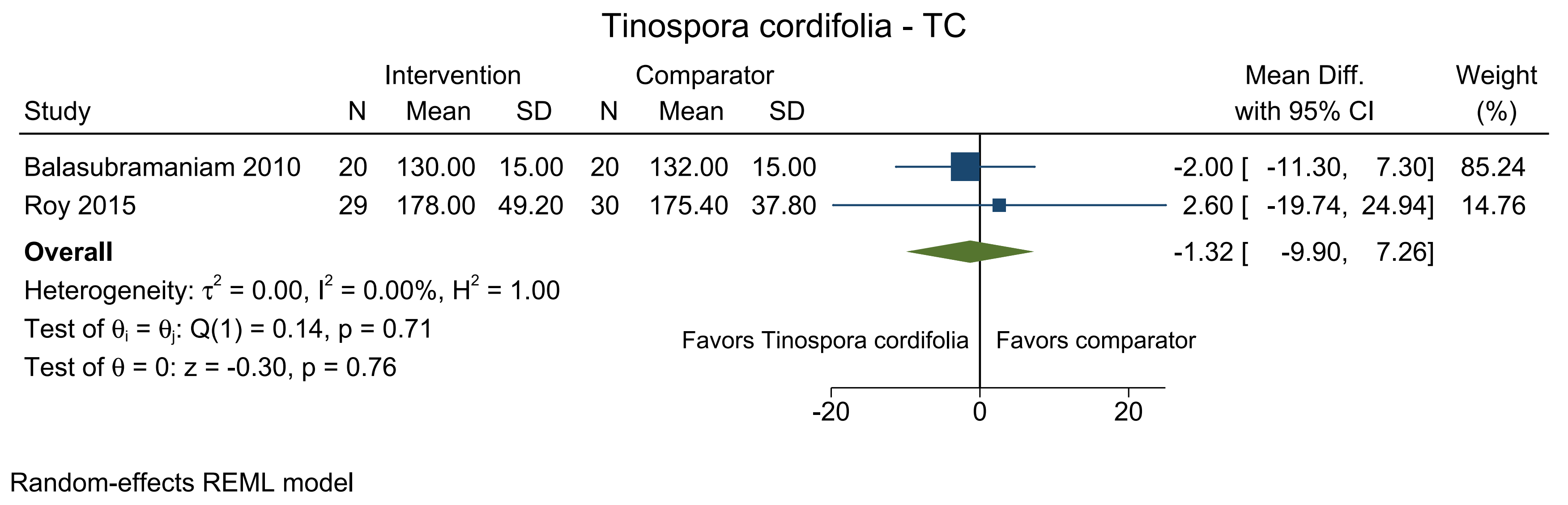
Tinospora cordifolia - TC
Intervention
Comparator
Study
N
Mean
SD
N
Mean
SD
Mean Diff. with 95% CI
Weight (%)
Balasubramaniam 2010 20 130.00 15.00 20 132.00 15.00 -2.00 [-11.30, 7.30] 85.24
Roy 2015 29 178.00 49.20 30 175.40 37.80 2.60 [-19.74, 24.94] 14.76
Overall -1.32 [-9.90, 7.26]
Heterogeneity: τ² = 0.00, I² = 0.00%, H² = 1.00
Test of θi = θj: Q(1) = 0.14, p = 0.71
Test of θ = 0: z = -0.30, p = 0.76
Favors Tinospora cordifolia
Favors comparator
-20
0
20
Random-effects REML model
